# Induced pluripotent stem cell-derived brain organoids as potential human model system for chemotherapy induced CNS toxicity

**DOI:** 10.3389/fmolb.2022.1006497

**Published:** 2022-09-15

**Authors:** Sophie Scholz, Karyn Lewis, Frederik Saulich, Matthias Endres, Wolfgang Boehmerle, Petra Huehnchen

**Affiliations:** ^1^ Klinik und Hochschulambulanz für Neurologie, Corporate Member of Freie Universität Berlin and Humboldt Universität zu Berlin, Charité—Universitätsmedizin Berlin, Berlin, Germany; ^2^ Department of Molecular and Experimental Nutritional Medicine, Institute of Nutritional Science, University of Potsdam, Nuthetal, Germany; ^3^ Molecular Genetics Group, Institute of Biology, Humboldt University of Berlin, Berlin, Germany; ^4^ NeuroCure Cluster of Excellence, Corporate Member of Freie Universität Berlin and Humboldt Universität zu Berlin, Charité—Universitätsmedizin Berlin, Berlin, Germany; ^5^ Berlin Institute of Health, Charité — Universitätsmedizin Berlin, Berlin, Germany; ^6^ Center for Stroke Research Berlin, Corporate Member of Freie Universität Berlin and Humboldt Universität zu Berlin, Charité—Universitätsmedizin Berlin, Berlin, Germany; ^7^ German Center for Neurodegenerative Diseases, Berlin, Germany; ^8^ German Center for Cardiovascular Research (DZHK), Partner Site Berlin, Berlin, Germany

**Keywords:** brain organoids, iPSC (induced pluripotent stem cell), chemotherapy, paclitaxel, neurotoxicity, apoptosis, 3R, new approach methodologies (NAM)

## Abstract

Neurotoxic phenomena are among the most common side effects of cytotoxic agents. The development of chemotherapy-induced polyneuropathy (CIPN) is a well-recognized adverse reaction in the peripheral nervous system, while changes of cognitive functions (post-chemotherapy cognitive impairment (PCCI)) are more diffuse and have only recently drawn scientific interest. PCCI in patients most often displays as short-term memory loss, reduced multitasking ability or deficits in language. Not least, due to a lack of preclinical human model systems, the underlying molecular mechanisms are poorly understood, and treatments are missing. We thus investigated whether induced pluripotent stem cell (iPSC)-derived brain organoids can serve as a human model system for the study of chemotherapy induced central nervous system toxicity. We robustly generated mature brain organoids from iPSC-derived neuronal precursor cells (NPC), which showed a typical composition with 1) dividing NPCs forming ventricle like structures 2) matured neurons and 3) supporting glial cells closer to the surface. Furthermore, upon stimulation the brain organoids showed functional signaling. When exposed to increasing concentrations of paclitaxel, a frequently used chemotherapy drug, we observed time dependent neurotoxicity with an EC50 of 153 nM, comparable to a published murine model system. Histological analysis after paclitaxel exposure demonstrated dose dependent apoptosis induction and reduced proliferation in the organoids with further Western blot analyses indicating the degradation of neuronal calcium sensor one protein (NCS-1) and activation of Caspase-3. We could also provide evidence that paclitaxel treatment negatively affects the pool of neuronal and astrocyte precursor cells as well as mature neurons. In summary our data suggests that human iPSC derived brain organoids are a promising preclinical model system to investigate molecular mechanisms underlying PCCI and to develop novel prevention and treatment strategies.

## 1 Introduction

Neurotoxic phenomena are among the most common adverse reactions of cytotoxic chemotherapy. Chemotherapy-induced polyneuropathy (CIPN) is a well-recognized toxic phenomenon in the peripheral nervous system (PNS) leading to degeneration of sensory neurons and their axons manifesting with painful par- and dysesthesia, numbness, fine motor deficits and ataxia (Boehmerle et al., 2015). In contrast to CIPN, cognitive function aberrations after chemotherapy [post-chemotherapy cognitive impairment (PCCI)]—also termed “chemobrain” or “chemofog” by the lay press—are lesser investigated and have only recently gained increasing scientific interest. Patients suffering from PCCI often report a decline in several cognitive domains such as verbal and visuo-spatial abilities, concentration, processing speed, attention span, and multitasking (Vardy et al., 2019). Although the brain is normally protected from neurotoxic substances by the blood-brain-barrier (BBB), PCCI was also observed in patients receiving chemotherapy with poor BBB-penetration capabilities such as paclitaxel as reviewed by Myers and coworkers (Myers et al., 2008). Despite its high clinical relevance—up to 50% of patients report a decline of cognitive function in temporal correlation to systemic chemotherapy, [reviewed by Vardy and Tannock (Vardy and Tannock, 2007)]—little scientific efforts are currently allocated to investigate the underlying pathomechanisms of PCCI. Among the proposed pathophysiological mechanisms contributing to cognitive deficits after chemotherapy are 1) mutations in the apolipoprotein E e4 allele (APOEe4) and p-glycoprotein, 2) DNA damage due to oxidative stress, 3) loss of neuroprotective hormones such as estrogen, 4) decreased adult hippocampal neurogenesis, 5) dysregulation of the immune system with release of proinflammatory cytokines and 6) chronic cerebral hypoxia due to anaemia and coagulopathies [as reviewed by Ahles and Saykin (Ahles and Saykin, 2007)]. The lack of preclinical research in PCCI and subsequently also the lack of newly identified molecular targets to treat and/or prevent PCCI are largely due to a deficit in existing preclinical (human) model systems. The advances made in the technology to generate and utilize induced pluripotent stem cells (iPSC) enable basic scientists to produce human, otherwise inaccessible tissue for research. We have previously used human iPSCs to differentiate iPSC-derived sensory neurons (DSN) and characterized iPSC-DSN as a new *in-vitro* model system for CIPN research. We were able to show that iPSC-DSN are a valuable translational tool to investigate molecular pathways of chemotherapy-induced neurodegeneration in the PNS as well as elucidate new potential disease markers and predictive factors for CIPN (Schinke et al., 2021; Huehnchen et al., 2022). Next to the advantage of a human model system, the iPSC technology presents the additive benefit to generate iPSC-derived neurons that stem from patients affected by the respective disease. While we could previously show that iPSC-derived neuronal precursor cells (NPC) are similarly susceptible to paclitaxel as murine neural stem cells (Huehnchen et al., 2017), which might contribute to PCCI by influencing hippocampal neurogenesis, modelling PCCI “in a dish” is far more complex. This is due to the three-dimensional structure of the brain and the underlying interplay of different cell types (neuronal precursors, mature neurons, astrocytes, oligodendrocytes, microglia, endothelial cells), which could all be differently affected by cytotoxic drugs and thereby contribute individually or together to PCCI (Renner et al., 2021). The recent establishment of organoids with their self-organization of progenitor cells into aggregates which form 3D organ-specific tissue (Lancaster et al., 2013) holds great potential to study mechanisms of disease as well as drug effects and/or toxicity. Brain organoids to date already share anatomical features with the human brain such as ventricle-like structures, progenitor and neuronal zones as well as glial populations (Pasca et al., 2015; Renner et al., 2017). While they have been used in the past to study treatment effects of chemotherapy on malignant brain tissue such as glioblastoma (Rybin et al., 2021), the effects of chemotherapy on a “healthy” brain organoid have not been investigated. Therefore, we investigated how paclitaxel—a microtubule-stabilizing agent that frequently causes neurotoxic phenomena in the peripheral and central nervous system leading to CIPN and PCCI—influences cell death and proliferation as well as the different cell types in brain organoids. Thus, determining whether iPSC-derived brain organoids can function as a potential translational *in-vitro* model system to study the pathomechanisms of PCCI.

## 2 Material and methods

### 2.1 Cell culture

#### 2.1.1 Brain organoid differentiation

Brain organoids were generated from human iPSC according to a previously published, but optimized protocol. All brain organoids originated from the established stem cell line BIHi250-A (https://hpscreg.eu/cell-line/BIHi250-A; Berlin Institute of Health Core Facility Stem Cells, Germany). For maintenance, human iPSCs were cultivated in essential 8 (E8) media (Stemcell Technologies, France) on Geltrex (Thermo Fisher, Waltham, MA) coated cell culture plates and colony passaged every 3–5 days using 0.5 M EDTA (Thermo Fisher). As a first step, human iPSCs were differentiated into NPSc as described previously (Huehnchen et al., 2017). In short, iPSCs were detached using TripLE select (Thermo Fisher) and single cell seeded in Geltrex coated 6-well plates (Corning Inc., Corning, NY) at a density of 0.5 × 10^6^ cells/well in E8 media supplemented with 10 µM Y-27632 (Stemcell Technologies) and kept in E8 media for 2 days with daily media exchange. Then the media was replaced with neural induction media (NIM), containing 10 µM SB431542 (Biogems, Westlake Village, CA) and 2 µM dorsomorphin (Biovision, Germany) for dual SMAD inhibition for another 4 days with daily media exchange. Differentiation into NPCs was assessed by the presence of typical NPC markers Sox1, Sox2, Nestin and Pax6 using immunocytochemistry and fluorescence activated cell sorting (FACS) analysis according to standard quality procedures. Cells were detached using TripLE select and frozen in Bambanker freezing medium (Nippon Genetics, Germany) supplemented with 10 µM Y-27632 á 3 × 10^6^ cells/vial until further usage. Three batches of NPCs were independently differentiated from iPSCs (≥3 passages apart), expanded and then frozen for later use to generate mature brain organoids. These three batches were treated as biological replicates (A/B/C) as proposed before (Chan and Teo, 2020).

**FIGURE 1:**
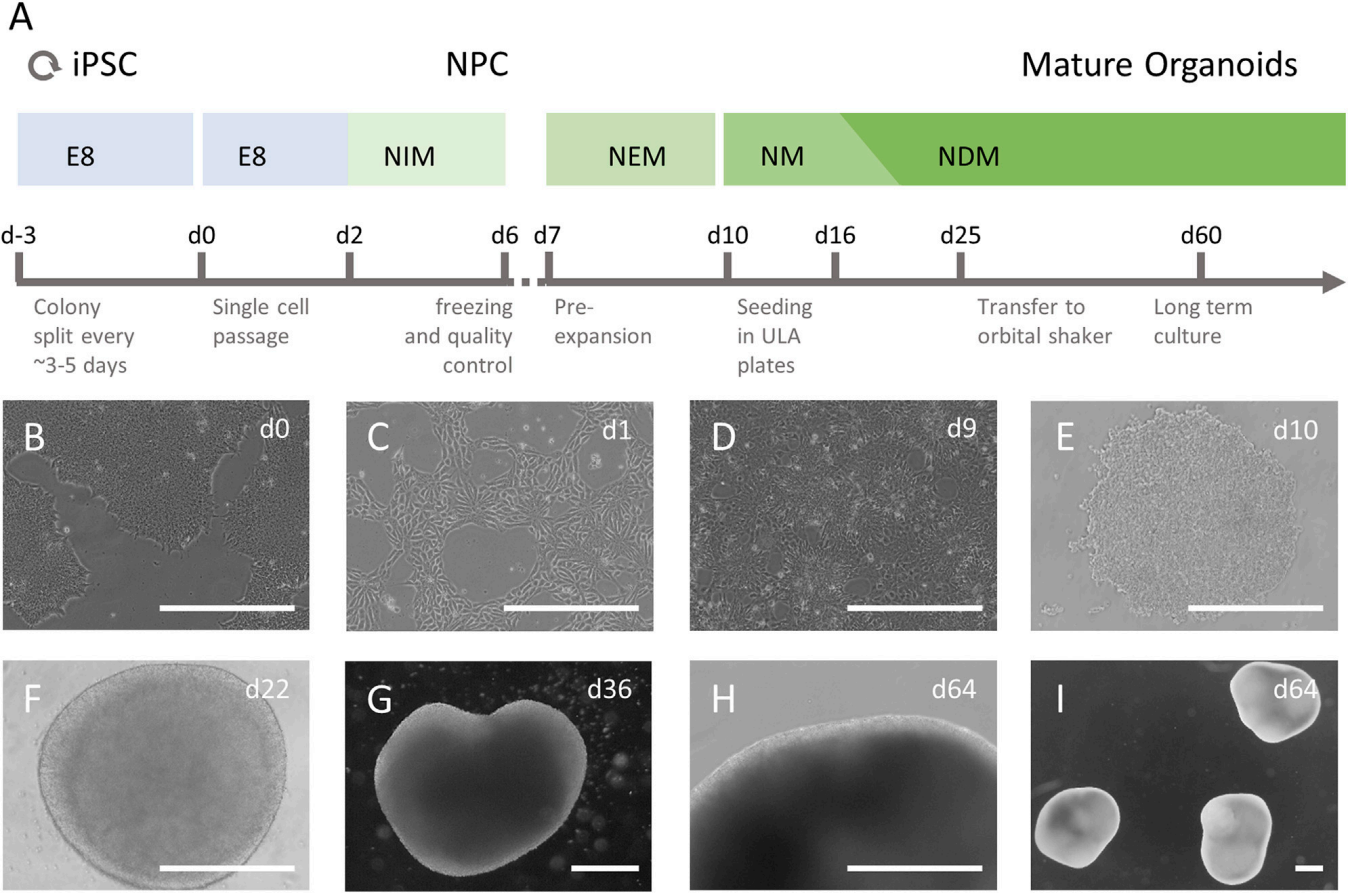
Culture of iPSC derived human brain organoids. **(A)** iPSCs were cultured in essential E8 media (E8) and after a single cell passage differentiated to neuronal progenitor cells (NPCs) in neural induction media (NIM), containing SB431542 and Dorsomorphin. Successful differentiation was assessed, and cells were frozen. Thawed NPCs were cultured and allowed to reach near confluency in neural expansion media (NEM) before being seeded in 96-well ultra-low attachment (ULA) plates. The cells were grown in neural medium (NM), containing hEGF and FGF basic from d10-d15, followed by neural differentiation medium (NDM), containing BDNF and NT3 until the end of culture. On day 25, immature organoids were transferred to 10 cm^2^ Petri dishes and kept on an orbital shaker at 65 rpm **(B–I)** Undifferentiated iPSC colonies **(B)** were single seeded **(C)** and differentiated to NPCs **(D)**. The NPCs were seeded in ULA plates as single cells **(E)** and after they formed a dense 3D aggregate with a smooth surface and grew in diameter **(F)** they were transferred to a dynamic culture system and allowed to mature **(G–I)**. Scale bars represent 500 µm.

Next, the protocol by Preusser et al. (2021) was optimized to generate self-organizing human brain organoids: vials of frozen NPCs were thawed in thawing medium (TM) containing 10 µM Y-27632 and pre-expanded in a Geltrex coated 6-well plate for 3 days in neural expansion media (NEM). Cells were detached using TripLE select and seeded at 3,000 cells/well in 100 µL into a 96-well round bottom ultra-low attachment plate (7007; Corning Inc.) in NEM supplemented with 10 µM Y-27632 followed by a short centrifugation of the plate (200–300 × g for 30–60 s). On the next day 100 µl neural medium (NM), containing 20 ng/ml human epidermal growth factor (hEGF; Peprotech, Germany) and 20 ng/ml fibroblast growth factor (FGF)-basic 154 a.a. (Peprotech), was added, followed by daily half medium exchange with NM for 5 days. Then the medium was slowly changed to neural differentiation medium (NDM), containing 20 ng/ml NT3 (Peprotech) and 20 ng/ml brain derived neurotrophic factor (BDNF; Peprotech), by changing half of the media every other day for 9 days with a stepwise increase of added media. Forming brain organoids were then transferred to 10 cm^2^ tissue culture dishes (Techno Plastic Products, Switzerland) and placed on an orbital shaker at 65 rpm until used for experiments, with half NDM media exchanges three times a week. Brain organoids that were later used for immunohistochemistry (IHC) analysis were kept in the 96-well plates with half media exchanges three times a week with NDM. A schematic overview of brain organoid generation is presented in Figure 1. For a detailed overview of all media please refer to the supplementary material (Supplementary Table S1).

#### 2.1.2 Paclitaxel treatment

Paclitaxel (CAS 33069-62-4; Adipogen, San Diego, CA) was diluted in DMSO (Sigma-Aldrich, Germany) to a stock solution of 8 mM fresh on each day of experiments and diluted three independent times for the treatment of each batch once. For the apoptosis induction assay independent stock solutions were prepared for each batch. Dilutions were prepared with NDM, the DMSO concentration was adjusted equally in each dilution to reach a final concentration of 0.125% in the well. Brain organoids were treated for 14 h with either NDM (medium control [MC]), NDM with 0.125% DMSO (vehicle control [VC]) or different concentrations, as stated per experiment, paclitaxel in 10 cm^2^ cell culture dishes on an orbital shaker to assure equal exposure, washed twice 5 min in fresh medium and then kept under normal culture conditions until being used for further experiments. All measurement or sampling time points always refer to the incubation period of 14 h plus the subsequent time in fresh media.

#### 2.1.3 Apoptosis induction assay

To assess the time-dependent dose-response of paclitaxel exposure on apoptosis induction in mature brain organoids, the RealTime-Glo™ Annexin V Apoptosis and Necrosis Assay was used (Promega, Germany). Apoptosis induction is measured by Annexin V binding to trans-localized phosphatidylserine, whereas necrosis is detected by a membrane-impermeable reagent binding to DNA. The sensitivity of the assay was previously tested by the company in 3D cultures (Data available on the company webpage). Furthermore, Annexin-V based assays have been previously used to assess cytotoxicity in 3D cultures (Xiao et al., 2020; Parseh et al., 2022). Three organoids per batch (day 58 in culture) were incubated with medium, vehicle or paclitaxel (1, 10, 100, 1,000, 10,000 nM) for 14 h, transferred to a white round bottom 96-well ultra-low attachment plate (MS-9096WZ; PHCbi, Netherlands) in NDM and the assay reagents were added according to the manufacturer’s instructions. Luminescence and fluorescence measurements were done 17, 18, 20, 22, 24, 26, 28, 32, 40 and 48 h after the start of the exposure with a SpectraMax iD3 plate reader (molecular devices, San Jose, CA) and normalized to the first measurement (17 h) as well as to the vehicle control. Effects of more than 20% compared to the VC were interpreted as relevant.

### 2.2 Western blot

For Western blot analysis one vial (3 × 10^6^ cells) of each batch of NPCs was thawed in TM, washed in 1 ml 1x PBS (−/−) (Thermo Fisher) and frozen in 1.5 ml Eppendorf tubes (Eppendorf, Germany) in liquid nitrogen. Protein expression for different lineage markers were analyzed in untreated immature brain organoids (day 36 in culture) and mature brain organoids (day 58 in culture). For analysis of acute effects of paclitaxel on protein expression, mature brain organoids (day 58 in culture) were treated for 14 h, transferred to fresh medium and collected either 10 h or 18 h later corresponding to 24 and 32 h time points respectively. To investigate long-term effects on cell type composition, mature brain organoids (day 58 in culture) were incubated with paclitaxel for 14 h and kept in culture for 6 days after the start of the exposure to assure clearance of dead cells as described by others before (Renner et al., 2021). Samples were cryo-grinded with EPPI-pestles (Schuett Biotec, Göttingen, Germany) and resuspended in RIPA-Buffer (50 mM Tris-HCl, 140 mM NaCl, 1% Triton-X-100, 1% Sodium deoxycholate, 0.1% sodium dodecyl sulphate supplemented with cOmplete™ Mini Protease Inhibitor Cocktail [Roche, France]) using 15 µl per organoid or 100 µl per vial NPCs. Protein concentration was measured with Pierce™ BCA Protein Assay Kit (Thermo Fisher) and samples were diluted to equal protein concentrations in RIPA Buffer. Prior to Western blot analysis, the linear detection range was investigated with pooled samples from mature brain organoids for all used markers by testing 1, 5, 10 and 15 µg of protein and one or two different dilutions of primary antibody. The protein amount and primary antibody concentration was selected based on these findings. Five µg (MAP2, SOX9, GFAP, Nestin, SOX2) or 10 µg (NeuN, cleaved caspase-3, NCS-1) per sample were loaded onto a polyacrylamide gel and separated by size. All samples from one batch, a protein ladder (LI-COR Biosciences, Germany) and a normalization sample (pooled from all batches) were loaded. Proteins were transferred wet to a PVDF membrane (Merck, Germany), which was blocked in Odyssey blocking buffer (LI-COR Biosciences) and incubated with primary antibodies overnight on ice on an orbital shaker, followed by washing and subsequent incubation with secondary antibodies for 1 h. Please refer to supplementary materials (Supplementary Table S2) for a detailed overview of all used antibodies and their dilutions. Fluorescence signals were detected on washed membranes with the Odyssey CLx system and bands were quantified using Image Studio V 5.2 analysis software (both LI-COR Biosciences) with automated background correction using upper/lower boundaries and median. Bands were normalized to the housekeeping proteins and to the normalization sample to make comparisons between blots possible. Effects of more than 20% compared to the VC were interpreted as relevant.

### 2.3 Histology

Brain organoids (day 64 or day 85 in culture) were washed in PBS, fixated in 4% paraformaldehyde overnight at 4°C and then dehydrated gradually in 0.4 and 0.8 M sucrose solution at 4°C. Brain organoids were then embedded in Tissue-Tek^®^ O.C.T.™ (Sakura, Japan) and snap frozen on dry ice for 2 min and then transferred to −80°C for long-term storage. 10 µm cryosections were made with a cryostat (Leica Biosystems, Germany), mounted on adhesion slides (Carl Roth, Germany) and kept at 4°C for no longer than a week.

Immunohistochemistry analyses were done using the same cell type markers as for western blot with additional markers for mature (MBP) and immature oligodendrocytes (Olig2). A detailed overview of all antibodies and dilutions can be referred to in the supplementary materials (Supplementary Table S2). The detection of each antigen in cryosections is described in the supplementary materials (Supplementary Table S3). Some sections underwent heat-induced antigen retrieval using a steam cooker (Braun, Germany), all were blocked with 10% normal goat serum (Abcam, United Kingdom) and permeabilized with either saponin (Sigma-Aldrich), Tween-20 (Sigma-Alrich) or Triton-X-100 (Carl Roth) followed by incubation with the primary antibody for 16 h or 40 h. After washing and secondary antibody incubation for 2 h, nuclei were stained using Qnuclear™ Deep Red Stain (Thermo Fisher) for 20–30 min. Sections were mounted with ProLong Gold (Thermo Fisher).

For analysis of proliferating cells, mature brain organoids (day 59 in culture) were treated with paclitaxel for 14 h, washed and 29 h after initiation of the exposure 10 µM of the thymidine analogue Bromodesoxyuridine (BrdU; Sigma-Aldrich) was added for 3 h. Organoids were embedded and sectioned as described above. Detection of BrdU in stainings included a heat-induced antigen retrieval with 0.1 M borate (Carl Roth) buffer and 0.05% Tween-20 at pH 8.5 for 20 min. IHC analysis, nuclear stain and mounting were performed as described above and additional experimental details are provided in the supplementary material (Supplementary Tables S2, S3). For analysis of DNA fragmentation, a terminal deoxynucleotidyl transferase dUTP nick end labeling (TUNEL) assay (Millipore, Burlington, MA,United States) was performed accordingly to the manufacturer’s instructions on sections of the same organoids as used for BrdU assay.

Microscopic slides were imaged within 2–3 days. For detection of fluorescence signals confocal images were taken using a Leica TCS SPE (Leica, Germany) laser scanning microscope equipped with 20x and 40x objectives, HC PL APO CS2 20x/0.75 DRY UV and HC PL APO CS2 40x/1.10 WATER UV respectively. Images were acquired with Leica Application Suite X (LAS X) Version 3.5.5. and processed with Open Microscopy Environment (OME) (Allan et al., 2012). Images from BrdU and TUNEL staining were analyzed semi-automatically using the ImageJ Analyse Particles function (Schneider et al., 2012). BrdU+ and TUNEL+ cell areas were summed and expressed as a percentage of cell nuclei and then normalized to vehicle control.

### 2.4 Calcium imaging

Brain organoids (day 86 in culture) were loaded with 5 µM Fluo-4 AM (Thermo Fisher) and 0.2% Pluronic™ F-127 (Thermo Fisher) in calcium imaging buffer (130 mM NaCl, 4.7 mM KCl, 1 mM MgSO_4_, 1.2 mM KH_2_PO_4_, 1.3 mM CaCl_2_, 20 mM Hepes and 5 mM glucose, pH 7.4) for 1 h in a cell culture incubator. Brain organoids were washed and transferred to 8-well µ-slides (ibidi, Germany) in 150 µl calcium imaging buffer and weighed down to prevent from moving. Fluorescence signals were detected every second for 141 s using a Leica TCS SPE laser scanning microscope equipped with a 20x objective, HC PL APO CS2 20x/0.75 DRY UV. Images were acquired with Leica Application Suite X (LAS X) Version 3.5.5. After 20 s of baseline measurement, 100 µl of a glutamate stock (250 μM; Sigma-Aldrich) was manually added to reach a final concentration of 100 µM in the well. Regions of interest (ROIs) in time series images were selected and analyzed using ImageJ (Schneider et al., 2012) and the change in fluorescence signals (ΔF/F) was calculated by: [F (signal at given time point) - F (mean of baseline signal)]/F (mean of baseline signal). Values above 0.2 ΔF/F were interpreted as a response to glutamate.

### 2.5 Statistics

All analysis of the data was done using R (R Core Team, 2021). Dose-response curves were modelled with the R package drc v3.0-1 (Ritz et al., 2015) and the EC50 and EC90 calculated. For any descriptive statistics the mean and standard deviation from three biological replicates were calculated, if not otherwise indicated. Statistical tests were only performed for the apoptosis induction assay, which had a sufficient dataset. The 95% confidence intervals were calculated using the predict function (interval = “confidence,” level = 0.95). Figures were made with the ggplot2 package (Wickham, 2016) depicting mean ± standard deviation and the individual data points of the biological replicates.

### 2.6 Data availability statement

The dataset that led to these results is available from the corresponding author at reasonable request.

## 3 Results

### 3.1 Characterization of human iPSC-derived brain organoids

The necessary steps and media used for brain organoid generation from human iPSCs are presented in a schematic overview in Figure 1A. Undifferentiated iPSC colonies were single seeded and differentiated to NPCs (Figures 1B–D). Before generation of brain organoids, NPCs were tested for marker expression and showed >90% positivity for relevant NPC markers such as Sox1, Sox2, Nestin and Pax6, which confirmed high purity of NPC cultures (data available on request). After seeding NPCs in ultra-low attachment plates, the cells began to self-organize in aggregates within a few hours (Figure 1E). Over the next 2 weeks, the brain organoids grew homogenously and became more circular with smooth bright edges that were consistent over the entire time of differentiation (Figure 1F). After the transfer to the dynamic culture system on day 25 in culture, brain organoids grew increasingly heterogeneous in shape and size (Figures 1G–I). In addition, a reduction in growth rate was observed the longer the brain organoids were in culture. All three independently generated batches of brain organoids differentiated and matured consistently in terms of three-dimensional aggregation, size, morphology and radial symmetry.

**FIGURE 2:**
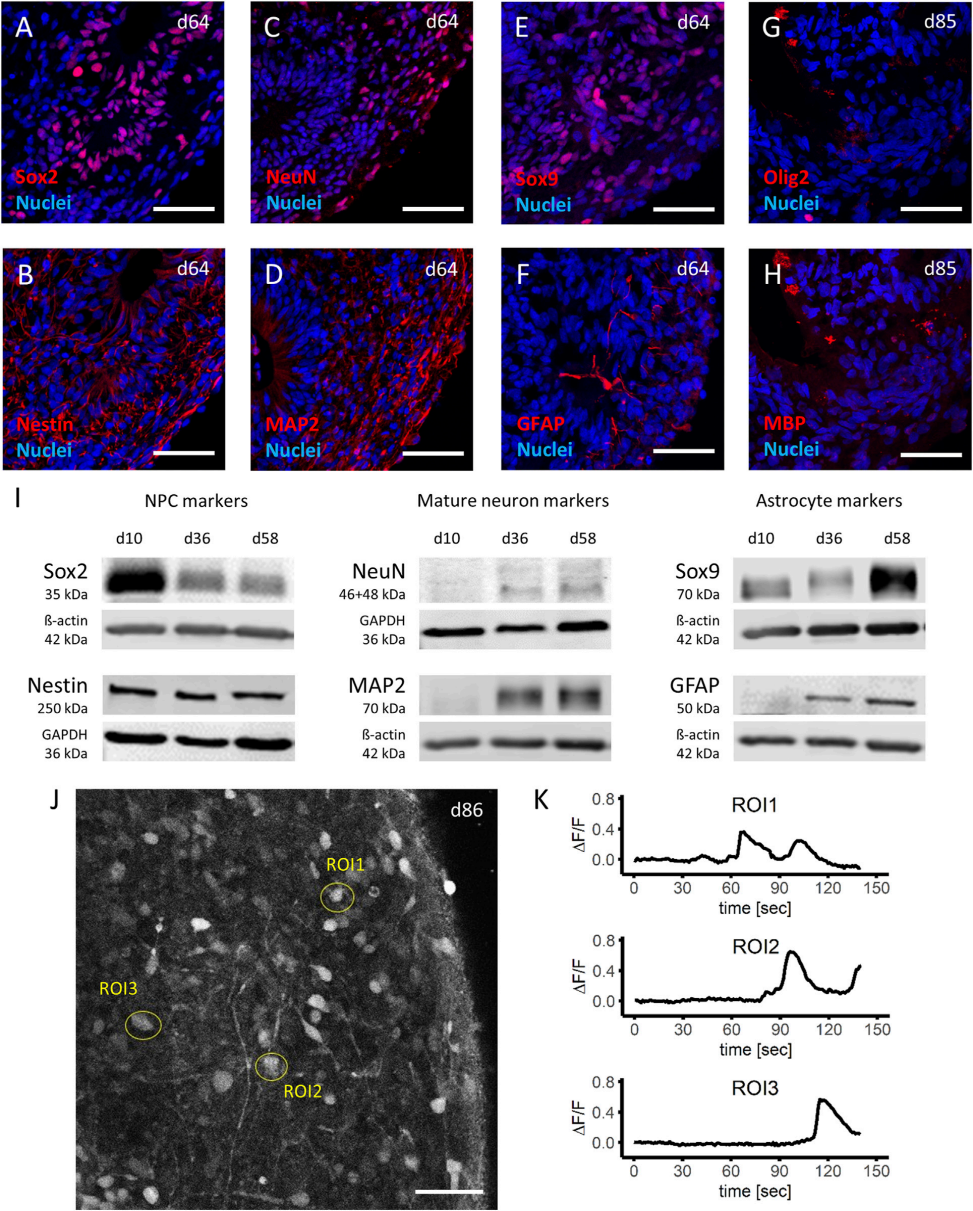
Characterization of mature brain organoids. **(A–H)** Immunohistochemistry analyses of important cell type markers in iPSC derived human brain organoids show the presence of neuronal progenitor cells (NPCs) by expression of Sox2 **(A)** and Nestin **(B)**, mature neuronal cells by NeuN **(C)** and MAP2 **(D)**, astrocytes by Sox9 **(E)** and GFAP **(F)** at day 64, as well as oligodendrocytes by Olig2 **(G)** and MBP **(H)** at day 85 of the differentiation protocol. **(I)** Western blot analyses of cell lysates from d10 (NPCs), d36 (early organoids) and d58 (late organoids), demonstrate a decrease of NPC markers (left panel), and an increase of mature neuron as well as astrocyte markers (middle panel and right panel, respectively) during the time of the differentiation protocol. **(J,K)** Stimulation of day 86 organoids with 100 µM glutamate after 20 s of baseline measurement and analyzation of three regions of interest (ROI) in live calcium imaging **(J)** demonstrated a depth dependent response, expressed as change in fluorescent signal (ΔF/F) **(K)**. Scale bars represent 50 µm.

In a next step, histologic analysis of mature brain organoids was carried out to assess organization and cell type composition within the organoids. In mature brain organoids, two different zones within the organoid can be distinguished based on anatomy and present cell types: a ventricular-like zone (VZ) and a cortical plate-like zone (CP). The VZ is characterized by the presence of Sox2-and Nestin-positive NPCs (Figures 2A,B; Supplementary Figures S1A,B) organized in rosette-like structures, while the CP can be identified by the presence of mature neurons (NeuN- and MAP2-positive, Figures 2C,D; Supplementary Figures S1C,D), astrocytes (Sox9-and GFAP-positive, Figures 2E,F; Supplementary Figures S2A,B) and oligodendrocytes (Olig2-and MBP-positive, Figures 2G,H; Supplementary Figures S2C,D), respectively. In line with the observed differentiation phases in the human brain as reviewed by Miller and Gauthier (Miller and Gauthier, 2007), oligodendrocytes matured last and were not present in organoids on day ∼60 but on day 85 in culture.

To confirm the histological findings, Western blot analysis performed at three different time points during the differentiation and maturation process of the brain organoids (day 10 [NPCs], day 36 and day 58 organoids) were included. Protein expression for markers of immature neurons Sox2 and Nestin was expectedly highest in NPCs at day 10 and in the case of Sox2 gradually decreased as brain organoids formed and were kept in culture (Figure 2I, left panel). Of note, even the older (day 58) brain organoids still expressed immature neuronal markers (particularly Nestin), which can be attributed to presence of NPCs in the ventricular-like zone of the brain organoid. While NPCs (day 10) did not express markers for mature neurons (NeuN and MAP2), both markers were detected in brain organoids (day 36 and onwards) with increasing expression (Figure 2I, middle panel). Similar results were observed for markers of astrocyte maturation (Sox9 and GFAP), which became increasingly expressed the longer the brain organoids were in culture (Figure 2I, right panel). These findings were replicated in brain organoids differentiated from three independent batches of NPCs and comparable results were observed. Furthermore, the western blot data confirmed the detection of the correct proteins found in histological analysis based on observed kDa.

Next, the functional state of the brain organoids was analyzed. Calcium imaging of brain organoids (day 85 in culture) was done from cells located at three different regions within the organoid (Figure 2J). Here, it was observed that after stimulation with 100 µM glutamate after 20 s of baseline recordings, the cytosolic calcium levels strongly increased. Not surprisingly, the location of the cells within the brain organoid with respect to the outer surface determined the time when the first increase in calcium was observed (ROI1: 45–67 s post stimulation, ROI2: 71–90 s post stimulation, ROI3: 93–112 s post stimulation; Figure 2K).

In conclusion, the optimized protocol reliably produced brain organoids derived from human iPSCs, which matured within 60–85 days and could produce limited responses to receptor activation as a first indicator of functional activity.

### 3.2 Cytotoxicity of human iPSC-derived brain organoids in response to chemotherapy

**FIGURE 3:**
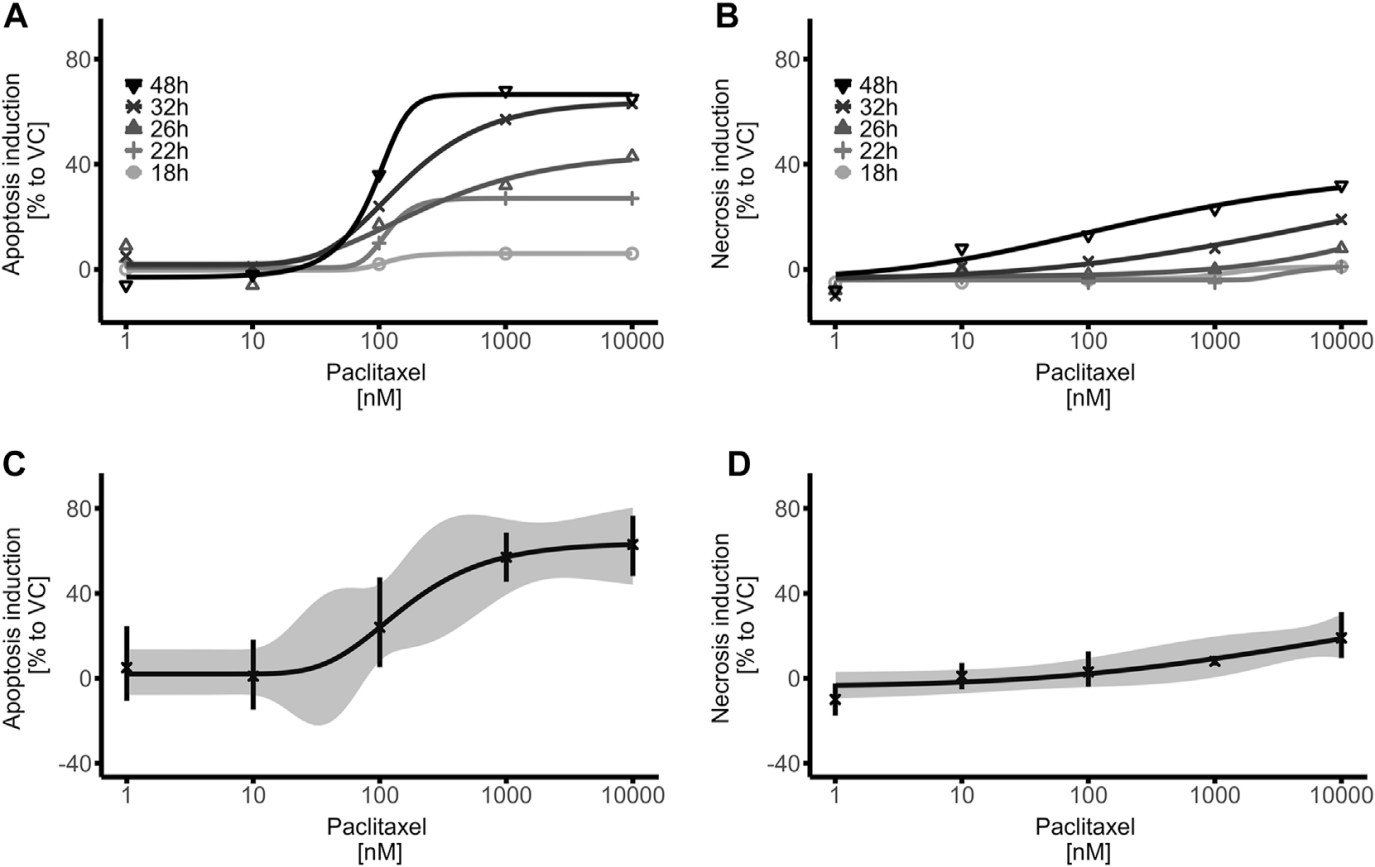
Time- and Dose-Response relationship of paclitaxel on apoptosis induction in mature brain organoids. **(A,B)** Brain organoids were treated with paclitaxel (1–10,000 nM) for 14 h, washed and apoptosis **(A)** and necrosis **(B)** induction was detected with RealTime-Glo™ Annexin V Apoptosis and Necrosis Assay (Promega) at different timepoints. **(C,D)** Dose Response curves of the 32 h measurement time point demonstrate relevant apoptosis induction (≥20% compared to VC) at concentrations as low as 100 nM **(C)**, while no relevant necrosis induction is measured **(D)**. Data were normalized to the first measurement (17 h) as well as to the vehicle control (VC) and represent means of three biological replicates with three technical replicates each; hence 9 organoids were treated equally per data point. The ribbon indicates the 95% confidence interval of the modeled curves, error bars represent standard deviation.

In a next step, it was investigated whether the iPSC-derived brain organoid model would respond to paclitaxel treatment in a time- and dose-dependent manner, as in murine and 2D culture models, determining the models use for toxicity investigations. Apoptosis and necrosis induction in human iPSC-derived brain organoids was assessed after exposure to increasing dosages of the microtubule-stabilizing agent paclitaxel (1–10,000 nM) and at different time points after the exposure period of 14 h. As expected, signs of apoptosis with increasing dosages of paclitaxel were observed, while the time after exposure also played an important role (non-linear regression models, Figure 3A; Supplementary Figure S3). The first relevant effects (≥20% compared to VC) on apoptosis induction in paclitaxel treated brain organoids were observed at the 22 h measuring time point, that is 8 h after the 14 h paclitaxel exposure period. At later measuring time points, such as 26, 32 and 48 h, the effects were greater and underline the impact of the post-incubation period on apoptosis induction. Expectedly, relevant necrosis induction (≥20% compared to VC) came after apoptosis induction and was first observed at the 40 h measuring time point, 26 h after the 14 h exposure period (non-linear regression models, Figure 3B; Supplementary Figure S4). The fact that the first relevant effects on necrosis induction were observed ∼20 h after binding of Annexin V to phosphatidylserine was detected, strongly supports cell death primarily via apoptosis and not necrosis. To avoid confounding effects of paclitaxel-induced apoptosis and secondary necrosis, the 32 h time point was chosen for further experiments, as strong apoptotic signals but no relevant necrotic signals were detected at that time point. The calculated EC50 for apoptosis induction, measured 32 h after start of the 14 h exposure with paclitaxel, was 153 nM while the EC90 was 1,084 nM (non-linear regression, Figure 3C). No statistically significant induction of apoptosis regardless of the time point was observed when cells were exposed to 10 nM of paclitaxel or less. For necrosis detection 32 h after induction of paclitaxel exposure, a calculation of the EC50 and EC90 was not possible, as the measured effects did not increase to relevant levels and no plateau within the chosen dose range and time point was detected (non-linear regression, Figure 3D). Calculated 95% confidence intervals and standard deviations for both apoptosis and necrosis induction were narrow and indicate a robust dataset and subsequent modeled dose-response-curves (Figures 3C,D). Based on these findings, the calculated EC50 and EC90 dosages of paclitaxel were used for further experiments.

In summary, paclitaxel was able to induce relevant apoptosis and necrosis in a time- and dose-dependent manner in mature brain organoids, indicating that human iPSC-derived brain organoids can function as 3D human model system to investigate cytotoxicity.

### 3.3 Possible mechanisms of paclitaxel-mediated cytotoxicity in human iPSC-derived brain organoids

**FIGURE 4:**
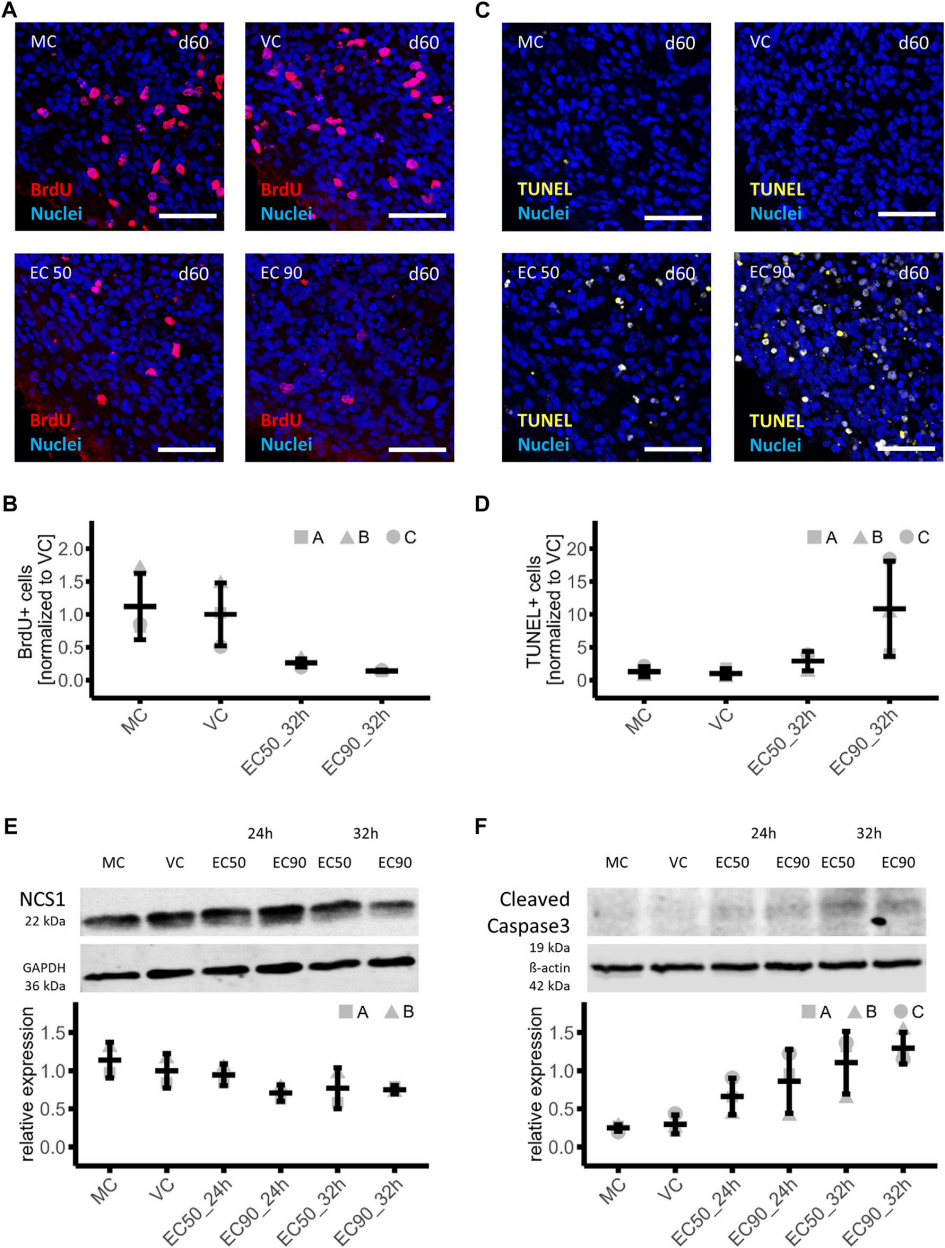
Effects of paclitaxel on cell proliferation, cell death and involved proteins in mature brain organoids. **(A,B)** Immunohistochemistry analyses of cell proliferation in brain organoids treated with paclitaxel (EC50: 153 nM and EC90: 1,084 nM) for 14 h with a subsequent culture in fresh media for 15 h and 3 h in BrdU containing medium (32 h sampling time point) or with medium (MC) or vehicle (VC) as controls **(A)**, demonstrated a 74% or 86% lower number of BrdU + cells in organoids treatedwith EC50 or EC90 respectively compared to the VC (*n* = 3) **(B)**. **(C,D)** Analysis of apoptosis via TUNEL assay in sections of the same brain organoids as used for proliferation assessment **(C)** showed a 2.9-fold and 10.9-fold increase in apoptotic cells in brain organoids treated with EC50 or EC90 paclitaxel respectively compared to the VC (*n* = 3) **(D)**. **(E,F)** Western blot analysis of organoids treated with paclitaxel for 14 h (EC50: 153 nM and EC90: 1,084 nM) with subsequent culture in fresh media for 10 h or 18 h (24 and 32 h sampling time points) show lower neuronal calcium sensor protein 1 (NCS-1) (*n* = 2) **(E)** and higher cleaved caspase 3 (*n* = 3) **(F)** abundance compared to the VC in a time- and dose-dependent manner. Histologic data are normalized to VC, Western blot data are normalized to a normalization sample. All data are shown in means and standard deviation.

Previously it was shown that a treatment with paclitaxel in clinically relevant dosages induces cognitive dysfunction in mice, which could be linked to a reduction of proliferating cells as well as a decrease of immature and mature neurons in the hippocampus (Huehnchen et al., 2017). Therefore, the incorporation of BrdU after exposure of mature brain organoids to vehicle, medium and 153 nM (EC50) as well as 1,084 nM (EC90) paclitaxel for 14 h, followed by a 18 h post incubation period (32 h sampling time point) was investigated. The number of BrdU + cells was quantified from histologic sections of the brain organoids (day 60 in culture). Cell proliferation was high and uniform in vehicle and medium control conditions demonstrated by large numbers of BrdU + cells in brain organoids. However, paclitaxel exposure induced a clear dose-dependent reduction of BrdU + cells, indicating severely reduced cell division (Figure 4A). The number of BrdU + cells was 74% or 86% lower after treatment with paclitaxel with EC50 or EC90 compared to the VC, respectively MC: 1.12 ± 0.50; VC: 1.00 ± 0.48; EC50: 0.26 ± 0.06; EC90: 0.14 ± 0.01 (Figure 4B).

Signs of apoptosis were also investigated in histologic sections from the same organoids that were used to investigate BrdU incorporation (day 60 in culture). The number of apoptotic nuclei was analyzed with fragmented DNA using the TUNEL method. An increased amount of TUNEL+ cells was observed in paclitaxel-treated brain organoids in a consistent manner throughout the brain organoid as compared to controls (Figure 4C). After treatment with the EC50, the amount of TUNEL+ cells was 2.9-fold and after EC90 treatment 10.9-fold increased compared to the VC (MC: 1.29 ± 0.79; VC: 1.00 ±; 0.74; EC50: 2.89 ± 1.48; EC90: 10.86 ± 7.24) (Figure 4D).

In a next step, it was investigated whether similar pathways for paclitaxel-induced apoptosis in brain organoids could be detected as were previously shown in murine neural stem cells or dorsal root ganglia neurons. A particular protein of interest in paclitaxel-induced neuronal apoptosis is the Neuronal calcium sensor 1 (NCS-1) protein. Paclitaxel enhances an interaction of NCS-1 with the Inositol-1,4,5-trisphosphate receptor (InsP3R), which results in a calcium efflux into the cytosol and triggers the activation of the calcium-dependent protease calpain. Calpain in turn cleaves NCS-1 as a negative feedback mechanism as well as triggers caspase-mediated apoptosis (Boehmerle et al., 2006; Boehmerle et al., 2007; Huehnchen et al., 2017). Therefore, protein expression of NCS-1 and cleaved caspase 3 were investigated in mature brain organoids treated with vehicle control (VC), medium (MC), 153 nM paclitaxel (EC50) or 1,084 nM paclitaxel (EC90) for 14h, with a subsequent culture in fresh media for 10 h or 18 h referred to as 24 and 32 h sampling time points respectively. Intensity of bands was normalized to housekeeping proteins and a normalization sample. Effects of more than 20% were interpreted as relevant. NCS-1 protein amount was 5%, 29%, 23% and 25% lower compared to VC after treatment with paclitaxel under different conditions: EC50 24 h, EC90 24 h, EC50 32 h, and EC90 32 h, respectively, indicating a relevant effect under all conditions but EC50 24 h (MC: 1.14 ± 0.23; VC: 1.00 ± 0.23; EC50 24 h: 0.95 ± 0.14; EC90 24 h: 0.71 ± 0.11; EC50 32 h: 0.77 ± 0.27; EC90 32 h: 0.75 ± 0.05; Figure 4E). These findings are in line with what has been found in neural murine cells suggesting that NCS-1 is degraded independent of the therapeutic mechanism of paclitaxel in human iPSC-derived brain organoids as well (Boehmerle et al., 2007). Assessment of the activation of caspase 3, a main executioner caspase in apoptosis (Nicholson et al., 1995), was done *via* Western blot using the same samples as for NCS-1 detection. Quantification of the 19 kDa subunit of cleaved caspase 3 demonstrate a time- and dose-dependent effect of paclitaxel induced activation of caspase 3 in brain organoids. Expression of cleaved caspase 3 was 2.2-fold, 2.9-fold, 3.7-fold and 4.3-fold increased compared to VC after treatment with paclitaxel under different conditions: EC50 24 h, EC90 24 h, EC50 32 h and EC90 32 h, respectively (MC: 0.25 ± 0.04; VC: 0.30 ± 0.12; EC50 24 h: 0.66 ± 0.24; EC90 24 h: 0.86 ± 0.42; EC50 32 h: 1.10 ± 0.41; EC90 32 h: 1.30 ± 0.21; Figure 4F).

In conclusion, a reduction in cell proliferation as well as an increase in apoptotic cells in our mature brain organoids was observed, which was corroborated by an increase of apoptotic proteins such as cleaved caspase-3 and alludes to the involvement of the NCS-1 pathway.

### 3.4 Effects of paclitaxel exposure on cell type markers in human iPSC-derived brain organoids

After demonstrating that signs of apoptosis and reduced cell proliferation can be detected in human iPSC-derived brain organoids after exposure to clinically relevant, nanomolar dosages of paclitaxel, it was examined how different cell types in the brain organoids might be affected by paclitaxel exposure. Protein abundance of multiple neuronal and glial lineage markers was assessed *via* semi-quantitative Western blot analyses in organoids treated for 14 h with either 153 nM (EC50) or 1,084 nM (EC90) paclitaxel, vehicle (VC) or medium control (MC), followed by subsequent culture in fresh media for 6 days. Intensity of bands was normalized to housekeeping proteins and a normalization sample. Effects of more than 20% were interpreted as relevant.

**FIGURE 5:**
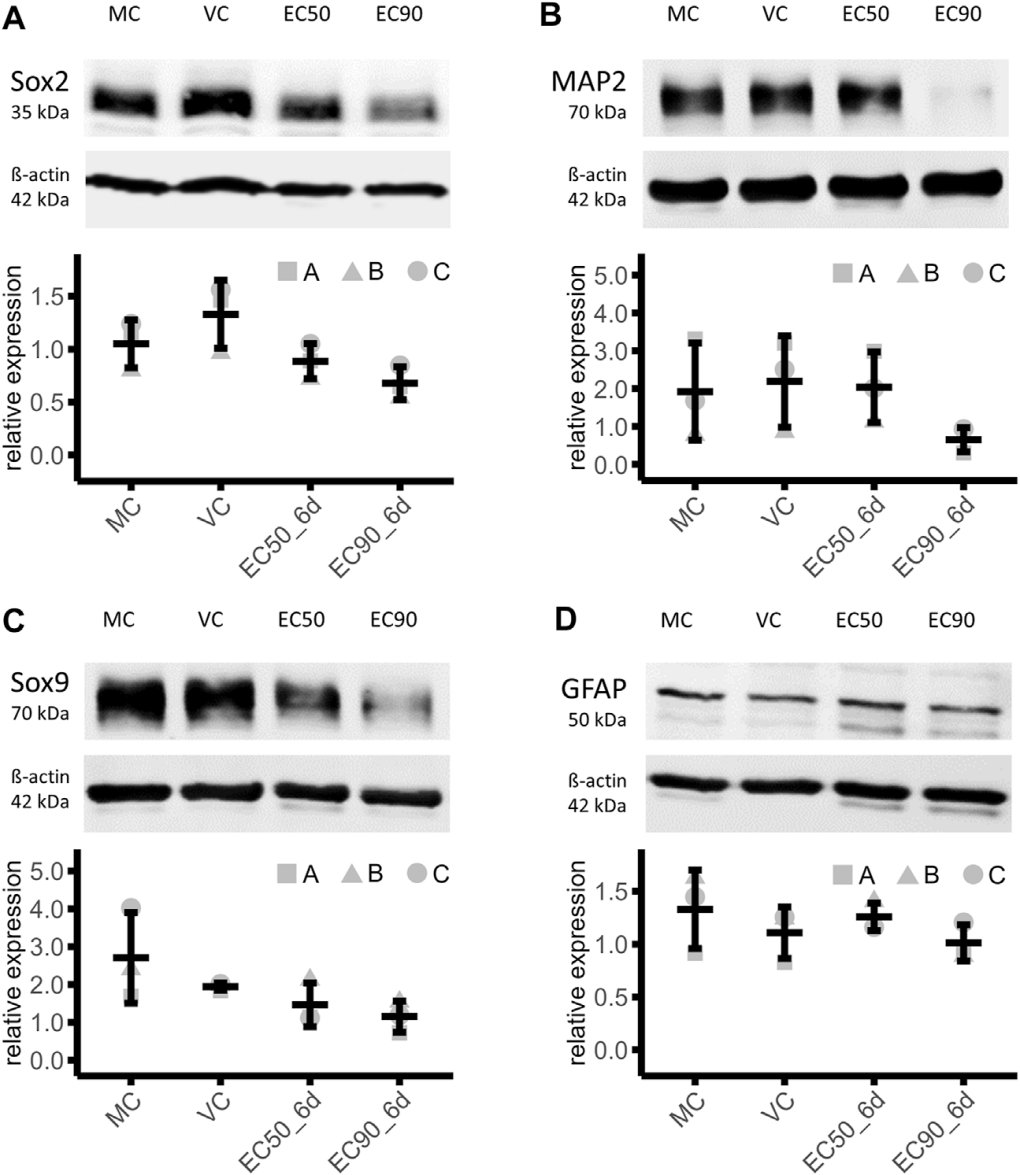
Effects of paclitaxel on cell type marker abundance. **(A–D)** Western blot analysis of organoids treated with paclitaxel for 14 h (EC50: 153 nM and EC90: 1,084 nM) with subsequent culture in fresh media for 6 days (6 days sampling time point) show lower abundance of neuronal precursor cell marker Sox2 **(A)**, mature neuronal marker MAP2 **(B)** and astrocyte precursor cell marker [Sox9] in paclitaxel treated organoids compared to the media (MC) and vehicle control (VC), while the mature astrocyte marker [GFAP] is not affected by paclitaxel treatment. Data are normalized to a normalization sample and shown in means and standard deviation (*n* = 3).

The NPC marker Sox2 was 33% lower in EC50 and 49% lower in EC90 treated organoids compared the VC (MC: 1.05 ± 0.23, VC: 1.33 ± 0.32, EC50: 0.89 ± 0.17, EC90: 0.68 ± 0.16), indicating a dose dependent effect of paclitaxel treatment on Sox2 expression in mature brain organoids (Figure 5A). Negative effects on neurons were demonstrated after treatment with the higher paclitaxel treatment, EC90. The abundance of mature neuronal marker MAP2 was 70% lower under this condition than in the VC. Treatment with EC50 did not result in a relevant effect (7% lower than VC) (MC: 1.93 ± 1.28, VC: 2.20 ± 1.21, EC50: 2.05 ± 0.93, EC90: 0.66 ± 0.32; Figure 5B). Further, the results demonstrate a negative dose-dependent effect of paclitaxel on Sox9 protein amount, a marker for astrocyte precursor cells. After treatment with the EC50 concentration, the amount of Sox9 was 25%, after EC90 treatment 41% lower than in the VC (MC: 2.70 ± 1.20, VC: 1.94 ± 0.09, EC50: 1.46 ± 0.58, EC90: 1.15 ± 0.41; Figure 5C). No relevant effects on the protein amount of GFAP were detected, a marker for mature astrocytes, after paclitaxel treatment (EC50: 13% higher and EC90: 9% lower than VC) (MC: 1.33 ± 0.37, VC: 1.11 ± 0.24, EC50: 1.26 ± 0.13, EC90: 1.01 ± 0.17; Figure 5D).

These effects were reproduced across all three batches of iPSC-derived brain organoids. In conclusion, relevant effects on protein level after paclitaxel treatment in both concentrations on NPC marker Sox2 and astrocyte precursor marker Sox9 were observed. Mature neuron marker MAP2 was only affected by the higher concentration of paclitaxel and mature astrocytic marker GFAP was not affected.

## 4 Discussion

Although the diagnosis of post-chemotherapy cognitive impairment still awaits addition to the International Statistical Classification of Diseases and Related Health Problems (ICD), overwhelming evidence from clinical trials and subsequent meta-analysis demonstrate the high medical need for patients undergoing treatment with cytotoxic drugs. To date there is still significant uncertainty regarding risk factors as well as the underlying pathophysiology and validated preventive or therapeutic options are completely lacking. This is in part due to a lack of suitable preclinical model systems. Although part of the disease can be modelled in rodents (Huehnchen et al., 2017; Nguyen et al., 2021), a model system with human cells suitable for both in-depth analysis of underlying pathways, as well as high-throughput screening is still lacking. Given the increasing relevance of iPSC-derived tissue models, iPSC-derived brain organoids as a human model system for the study of chemotherapy induced central nervous system toxicity were investigated. Results have shown that with this optimized protocol iPSC-derived brain organoids can reliably be produced, which after prolonged culture (60–85 days) consist of ventricle-like structures and cortical-plate like structures. Next to NPCs, mature neurons, astrocytes and oligodendrocytes were detected indicating the maturation state of the brain organoids. Additionally, brain organoids showed functional calcium signalling upon stimulation. These observations replicate and expand previous findings made in this model (Pasca et al., 2015; Preusser et al., 2021). Although brain organoids represent the composition of human brain tissue to some extent, one limitation of the protocol used here especially for immunological questions is the lack of microglia. Other groups tried to address this limitation and iPSC-derived brain organoid models with microglia are being developed (Xu et al., 2021). The present work investigates chemotherapy-induced cytotoxicity and mechanisms of apoptosis, secondary neuro-immune interactions—for which microglia are an essential part—are thus less relevant. Another limitation of iPSC-derived brain organoids used in this study is the lack of functional blood vessels and a blood brain barrier. This is of particular relevance as paclitaxel is a lipophilic drug able to passively diffuse into the brain where it is quickly eliminated by a p-glycoprotein mediated mechanism *in vivo* (Breedveld et al., 2006). To mitigate this limitation, exposure was adapted to measured paclitaxel levels in rodent brains (Huehnchen et al., 2017). Building on this data, very low concentrations of paclitaxel and a short incubation time was used to mimic the situation with an intact blood brain barrier.

Interestingly, when mature iPSC-derived brain organoids were exposed to increasing concentrations of paclitaxel, time-dependent neurotoxicity with an EC50 comparable to published murine model systems could be observed (Boehmerle et al., 2006; Huehnchen et al., 2017). Furthermore, histological analysis of paclitaxel-treated organoids demonstrated dose-dependent apoptosis induction and reduced cell proliferation, which was previously described in a murine model of PCCI (Huehnchen et al., 2017). Preliminary data from Western blot analysis also indicate that similar cell death mechanisms (i.e., NCS-1 and apoptotic cascades) as described before are relevant in iPSC-derived brain organoids, which underlines the usefulness of this novel model system. The effect of paclitaxel on NCS-1 expression was not as strong as previously demonstrated, possibly due to the presence of many different cell types with differential susceptibility to paclitaxel in the brain organoid system. Therefore, effects from Western blot analysis regarding NCS-1 in brain organoids are potentially diluted and not as pronounced as effects measured in pure cultures such as NPCs or dorsal root ganglion neurons (Boehmerle et al., 2007).

On the other hand, however, the presence of many different neuronal and glial cell types provides an advantage of the brain organoid model system over other 2D models as the effects of paclitaxel on specific cell types can be studied. Results have shown, that paclitaxel treatment negatively affects the pool of neuronal and astrocyte precursor cells as well as mature neurons in high concentrations, while limited effects on mature astrocytes were observed. The long-term effect on neuronal precursor cells was not as distinct as expected after the finding of an acute reduction in cell proliferation of almost 90%. The cell proliferation assay raise the possibility that neural stem cells/NPCs as the cell type with the highest proliferative capacity are primarily targeted as previously demonstrated (Huehnchen et al., 2017; Park et al., 2021). It is likely, however, that although neural stem cells are quite susceptible to paclitaxel treatment, they recover to a certain degree within the 6 days post treatment period. Nevertheless, even transient effects on NPCs may lead to a reduction in mature neurons as the differentiation process is interrupted. Evaluation of the cell types most vulnerable to cytotoxic treatments in this model is challenging as a non-uniform distribution of cell types across different regions was observed. This finding poses a high risk for potential bias when only a few sections of the organoids are analysed. To reduce bias, western blot analysis of entire organoids as a less sensitive but also less biased read-out was therefore chosen. The obvious caveat of this approach is that spatial resolution is lost and no information on a single cell level can be obtained. A way to overcome this issue in future studies would be to make serial sections of entire organoids and to image and evaluate entire stacks instead of only parts. This approach should be hence considered in future studies using this model. Significant effects on MAP2 expression were only observed with higher paclitaxel dosages (EC90). This raises the question of whether paclitaxel’s effects on calcium homeostasis, to which paclitaxel-induced neurotoxicity was attributed to in NPCs and dorsal root ganglia neurons in the lower dose ranges (30–150 nM), is also the main contributor to cell death in the higher dose range or whether paclitaxel’s main mechanism of action (i.e., hyperstabilization of microtubules) is also of relevance. While the latter leads to inhibition of axonal transport and thereby disruption of neuronal functionality and eventually cell death, the doses needed to induce these effects are much higher around 10–200 µM (Nakata and Yorifuji, 1999; LaPointe et al., 2013), which is still at least one order of magnitude higher than the highest dose we used in our experiments (EC90 ∼1 µM). Therefore, while it cannot be ruled out that paclitaxel induces hyperstabilization of microtubules even at 1 µM and thereby contributes to the observed decrease in MAP2 expression, it is less likely. Another interesting finding is the reduced marker expression of Sox9 after paclitaxel treatment, while protein expression of GFAP remained unchanged. Sox9 is expressed in different immature cells and a main transcription factor that triggers astro-gliogenesis (Kang et al., 2012; Klum et al., 2018). A decreased expression of Sox9 without differences in expression of mature astrocytes could also be interpreted as a reduced activation of Sox9. As paclitaxel-induced cytotoxicity towards NPCs was observed and it was previously shown that (hippocampal) neurogenesis is reduced in paclitaxel-treated mice, a reduced activation of Sox9 might be due to enhanced neurogenesis as normally Sox9 activation is critical for suppression of neurogenesis and a subsequent shift towards gliogenesis (Vong et al., 2015). In line with findings of this study, a reduced number of glial precursor cells was demonstrated in brain sections of children receiving various treatments with chemotherapy. Additionally, the authors investigated effects of the chemotherapeutic agent methotrexate in detail in a rodent model and were able to reproduce the effects observed in patients (Gibson et al., 2019). Similar effects of paclitaxel on glial precursor cells, were demonstrated in a 2D model of neuronal and glia differentiation from mouse neural stem cells. Treatment of cells with 50 nM paclitaxel resulted in a reduced number of glial precursor cells and mature oligodendrocytes, but not of mature astrocytes or neurons (Park et al., 2021). These effects on cell type changes induced by paclitaxel could be one factor contributing to PCCI, and have already been reviewed in this context by Nguyen and Ehrlich (Nguyen and Ehrlich, 2020).

In summary, it was demonstrated that human iPSC-derived brain organoids are a promising model to investigate CNS toxicity as they combine many features murine models or 2D culture models are lacking. In terms of post-chemotherapy cognitive impairment, iPSC-derived brain organoids present the opportunity to elucidate the underlying pathomechanism by studying cells from patients with severe PCCI and the interplay between different cell types as well as how they contribute to PCCI.

## Data Availability

The original contributions presented in the study are included in the article/Supplementary Material, further inquiries can be directed to the corresponding author.
