# Supplementary material for: Induced pluripotent stem cell-derived brain organoids as potential human model system for chemotherapy induced CNS toxicity
Source: Front Mol Biosci. 2022 Sep 15;9:1006497. doi: 10.3389/fmolb.2022.1006497 (PMC9520921; doi:10.3389/fmolb.2022.1006497)
Supplement: Supplementary file 1 [file DataSheet1.DOCX]

Supplementary Material

# Supplementary Figures and Tables

## Supplementary Figures

### Neural progenitor cell and mature neuronal markers

### Supplementary Figure 1. Immunohistochemistry analyses of important cell type markers in iPSC derived human brain organoids show the presence of neuronal progenitor cells (NPCs) by expression of Sox2 [A] and Nestin [B] and mature neuronal cells by NeuN [C] and MAP2 [D] at day 64 of the differentiation protocol. Scale bars represent 50µm.


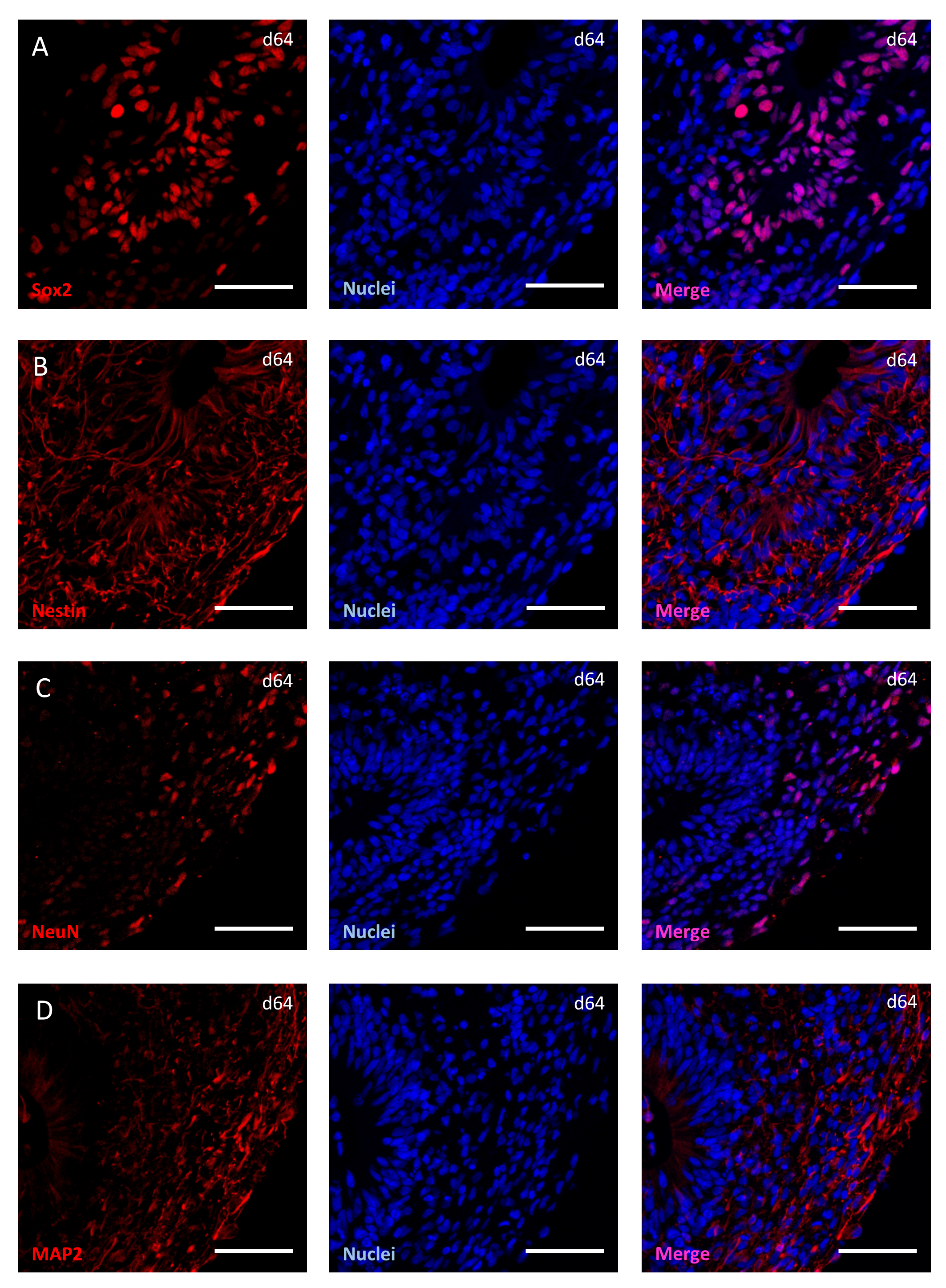


### Astrocyte and oligodendrocyte markers


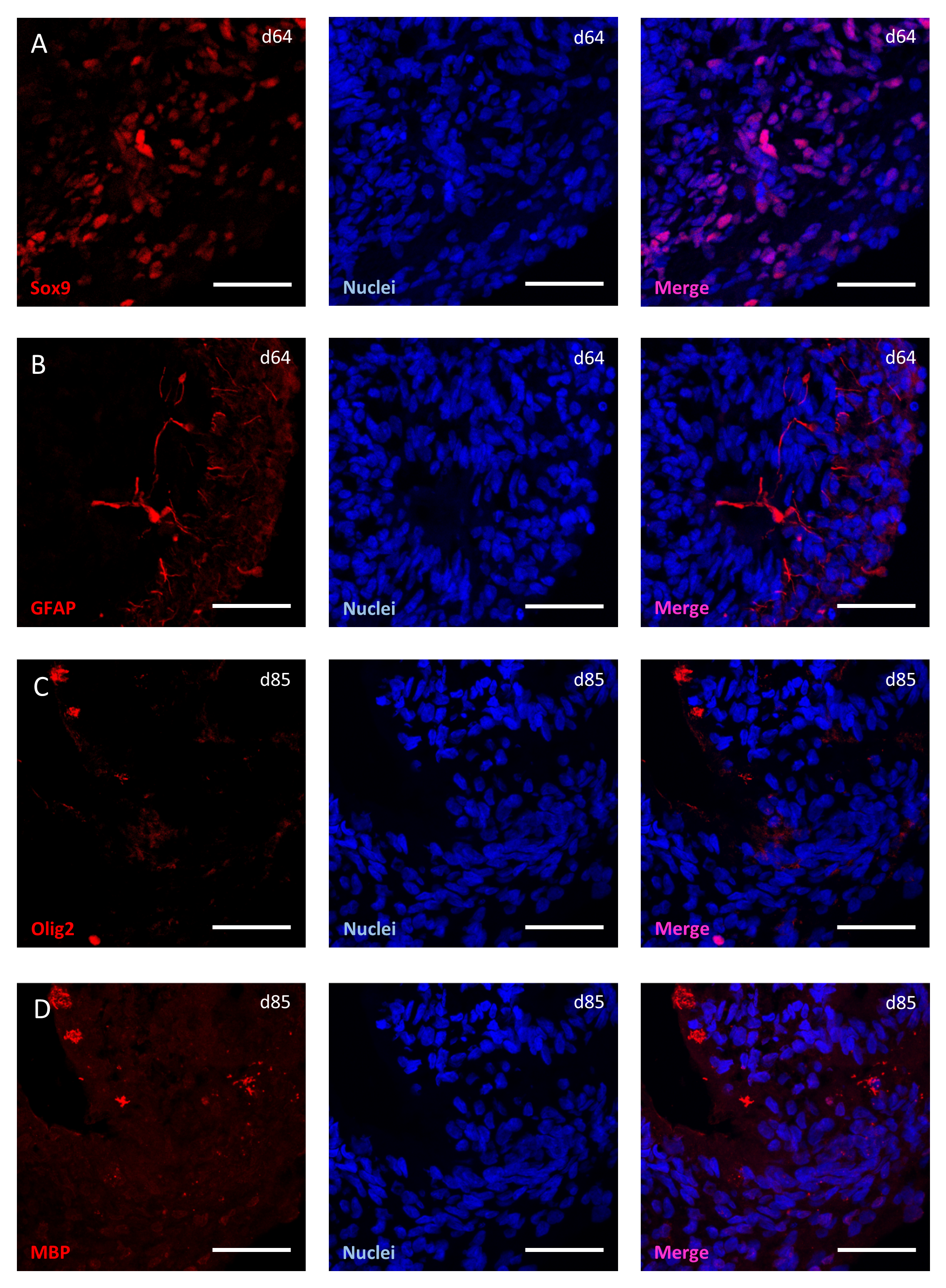


**Supplementary Figure 2.** Immunohistochemistry analyses of important cell type markers in iPSC derived human brain organoids show the presence of astrocytes by Sox9 [A] and GFAP [B] at day 64, as well as oligodendrocytes by Olig2 [C] and MBP [D] at day 85 of the differentiation protocol. Scale bars represent 50µm.

### Time-Dose-Response relationship of paclitaxel on apoptosis induction in mature brain organoids


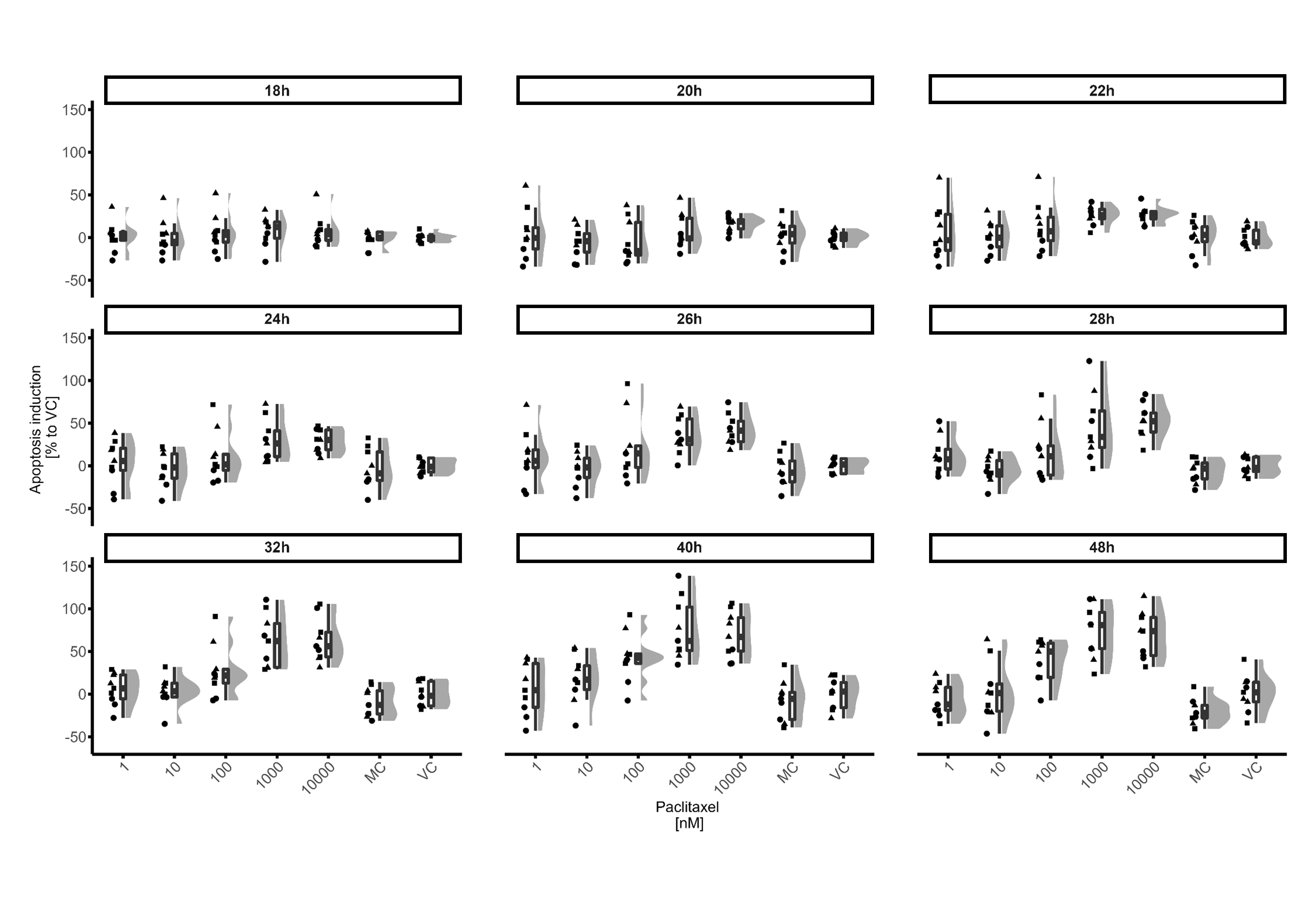


### Supplementary Figure 3. Brain organoids (day 58 of the differentiation protocol) were treated with paclitaxel (1-10000 nM) a vehicle (VC) or a medium control (MC) for 14h, washed, and apoptosis induction was detected via Annexin V binding to trans-localized phosphatidylserine with RealTime-Glo™ Annexin V Apoptosis and Necrosis Assay (Promega) at different time points (17 h-48 h post initiation of the incubation period). Data were normalized to the first measurement (17h) as well as to the vehicle control (VC). Raincloud plots were used to visualize the dataset. Data points represent measurements of all three batches (square: A; triangle: B; circle: C); boxblots show the median, hinges show the first and third quartiles and whiskers show the extend from the hinge to the largest value no further than 1.5 * interquartile range from the hinge; flat violins show the distribution of the data.

### Time-Dose-Response relationship of paclitaxel on necrosis induction in mature brain organoids


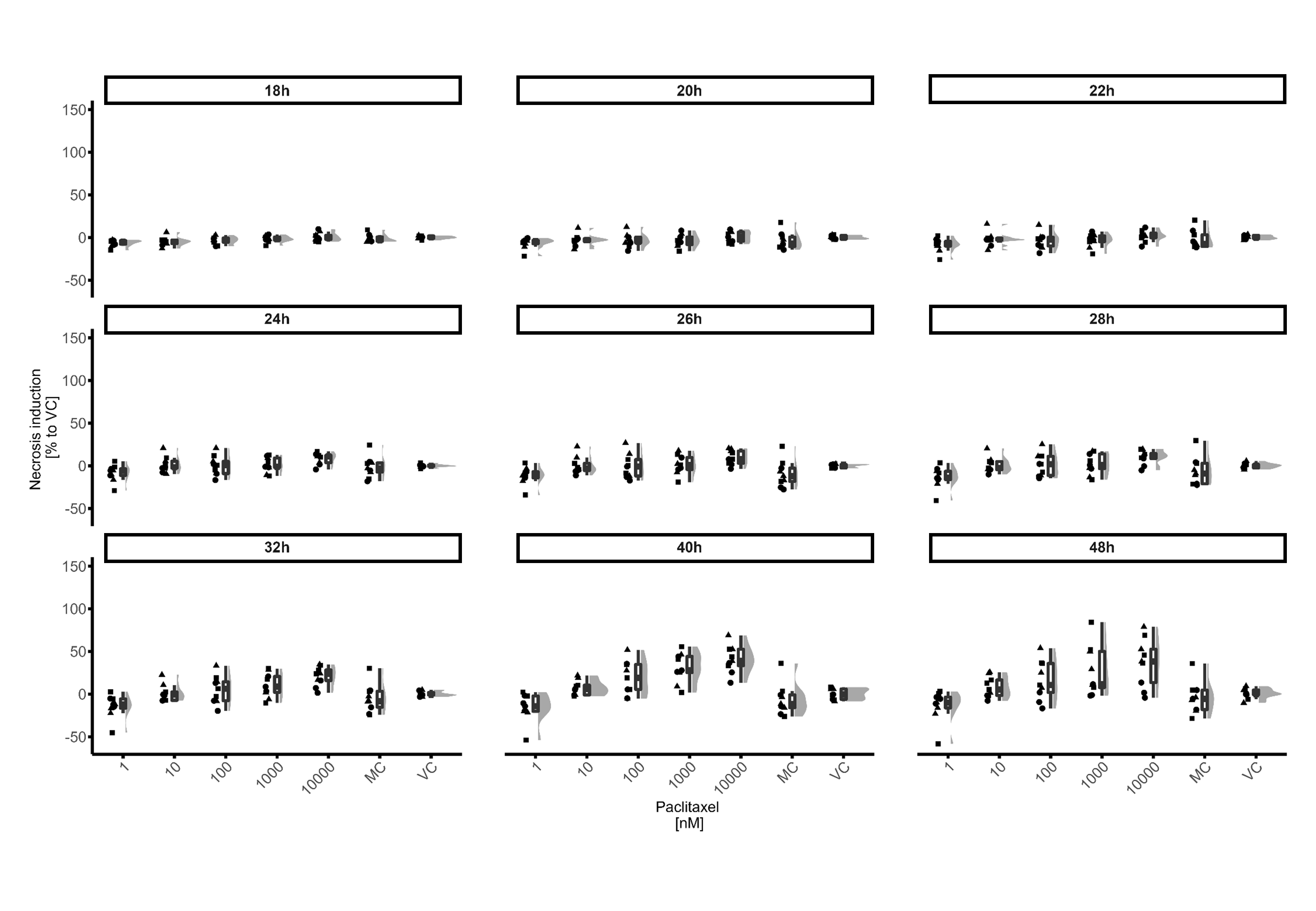


**Supplementary Figure 4.** Brain organoids (day 58 of the differentiation protocol) were treated with paclitaxel (1-10000 nM) a vehicle (VC) or a medium control (MC) for 14h, washed, and necrosis induction was detected via a membrane impermeable reagent binding to DNA with RealTime-Glo™ Annexin V Apoptosis and Necrosis Assay (Promega) at different time points (17 h-48 h post initiation of the incubation period). Data were normalized to the first measurement (17h) as well as to the vehicle control (VC). Raincloud plots were used to visualize the dataset. Data points represent measurements of all three batches (square: A; triangle: B; circle: C); boxblots show the median, hinges show the first and third quartiles and whiskers show the extend from the hinge to the largest value no further than 1.5 * interquartile range from the hinge; flat violins show the distribution of the data.

## Supplementary Tables

### Media composition

**Supplementary Table 1.** Composition of media used in cell culture.

| Component | Supplier | concentration | | stock solution | | 100 | | mL |
| --- | --- | --- | --- | --- | --- | --- | --- | --- |
| **Essential 8 media (E8**) | | | | | | | | |
| TeSR E8 Basal Media | Stemcell Technologies 05991 | 96 | % | 100 | % | | 96 | mL |
| TeSR E8 Supplement | Stemcell Technologies 05992 | 1 | x | 25 | x | | 4 | mL |
| **Neural induction media (NIM)** | | | | | | | | |
| DMEM/F12 | Thermo Fisher 11330-032 | 95.96 | % | 100 | % | | 95.96 | mL |
| B27 Supplement | Thermo Fisher 175047-044 | 1 | x | 50 | x | | 2 | mL |
| N2 Supplement | Thermo Fisher 17502-048 | 1 | x | 100 | x | | 1 | mL |
| Dorsomorphin | Biovision 1686-5 | 2 | µM | 10000 | µM | | 0.02 | mL |
| SB431542 | Biogems 3014193 | 10 | µM | 50000 | µM | | 0.02 | mL |
| P/S | Thermo Fisher 15140-122 | 100 | U/mL | 10000 | U/mL | | 1 | mL |
| **Thawing media (TM)** | | | | | | | | |
| KO DMEM/F12 | Thermo Fisher 12660-012 | 99.8 | % | 100 | % | | 99.8 | mL |
| RI Y-27632 | Stemcell Technologies 72304 | 10 | µM | 5000 | µM | | 0.2 | mL |
| **Neural expansion media (NEM)** | | | | | | | | |
| Neurobasal Media | Thermo Fisher 21103-049 | 48.4 | % | 100 | % | | 48.4 | mL |
| Advanced DMEM/F12 | Thermo Fisher 12634-010 | 48.4 | % | 100 | % | | 48.4 | mL |
| NI-Supplement | Thermo Fisher A16477-01 | 1 | x | 50 | x | | 2 | mL |
| P/S | Thermo Fisher 15140-122 | 100 | U/mL | 10000 | U/mL | | 1 | mL |
| **Neural media (NM)** | | | | | | | | |
| Neurobasal Media | Thermo Fisher 21103-049 | 95.9 | % | 100 | % | | 95.9 | mL |
| Glutamax | Thermo Fisher 35050-061 | 1 | x | 100 | x | | 1 | mL |
| B27 Supplement | Thermo Fisher 175047-044 | 1 | x | 50 | x | | 2 | mL |
| hEGF | Peprotech AF-100-15 | 20 | ng/mL | 40000 | ng/mL | | 0.05 | mL |
| FGF Basic 154 a.a | Peprotech 100-18B | 20 | ng/mL | 40000 | ng/mL | | 0.05 | mL |
| P/S | Thermo Fisher 15140-122 | 100 | U/mL | 10000 | U/mL | | 1 | mL |
| **Neural differentiation media (NDM)** | | | | | | | | |
| Neurobasal Media | Thermo Fisher 21103-049 | 95.96 | % | 100 | % | | 95.96 | mL |
| Glutamax | Thermo Fisher 35050-061 | 1 | x | 100 | x | | 1 | mL |
| B27 Supplement | Thermo Fisher 175047-044 | 1 | x | 50 | x | | 2 | mL |
| BDNF | Peprotech AF-450-02 | 20 | ng/mL | 100000 | ng/mL | | 0.02 | mL |
| NT3 | Peprotech AF-450-03 | 20 | ng/mL | 100000 | ng/mL | | 0.02 | mL |
| P/S | Thermo Fisher 15140-122 | 100 | U/mL | 10000 | U/mL | | 1 | mL |

### Antibodies used in Western blot and immunohistochemistry analyses

**Supplementary Table 2.** Overview of all primary and secondary antibodies used as well as dilution factors.

| Antibodies | Use in Western blot | Use in Immunohistochemistry |
| --- | --- | --- |
| ms-a-Nestin Abcam, ab22035, RRID:AB_446723 | 1:5000 | 1:100 |
| rb-a-Sox2 Abcam, ab97959, RRID:AB_2341193 | 1:5000 | 1:100 |
| rb-a-GFAP Agilent, Z0334, RRID:AB_10013382 | 1:5000 | 1:145 |
| rb-a-Sox9 Abcam, ab185966, RRID:AB_2728660 | 1:5000 | 1:123 |
| ms-a-NeuN Millipore, MAB377, RRID:AB_2298772 | 1:5000 | 1:100 |
| rb-a-MAP2 abcam, ab32454, RRID:AB_776174 | 1:5000 | 1:100 |
| ms-a-NCS-1 Santa Cruz Biotechnology, sc-376206, RRID:AB_11008074 | 1:1000 | - |
| rb-a-Cleaved caspase 3 Cell Signaling Technology, 9661, RRID:AB_2341188 | 1:5000 | - |
| ms-a-MBP Thermo Fisher Scientific, MA5-15922, RRID:AB_11154789 | - | 1:200 |
| rb-a-Olig2 Millipore, AB9610, RRID:AB_570666 | - | 1:200 |
| rat-Anti BrdU, Abcam, ab6326, RRID:AB_305426 |  | 1:500 |
| rb-a-GAPDH 1:5000 (Cell Signaling Technology, 2118, RRID:AB_561053 | 1:5000 | - |
| ms-a-β-actin 1:10000 (Cell Signaling Technology, 3700, RRID:AB_2242334 | 1:10000 | - |
| IRDye 800CW Donkey a-ms (LI-COR Biosciences, 926-32212, RRID:AB_621847 | 1:15000 | - |
| IRDye 800CW Donkey a-rb (LI-COR Biosciences, 926-32213, RRID:AB_621848 | 1:15000 | - |
| IRDye 680RD Donkey a-ms (LI-COR Biosciences, 926-68072, RRID:AB_10953628 | 1:15000 | - |
| IRDye 680RD Donkey a-rb (LI-COR Biosciences, 926-68073, RRID:AB_10954442 | 1:15000 | - |
| goat-a-ms-568 (Thermo Fisher Scientific, A-11031, RRID:AB_144696) | - | 1:400 |
| goat-a-rb-488 (Thermo Fisher Scientific, A-11034, RRID:AB_2576217) | - | 1:400 |
| goat-a-ms-488 (Thermo Fisher Scientific, A-11029, RRID:AB_2534088) | - | 1:500 |
| goat-a-rb-568 (Thermo Fisher Scientific, A-11036, RRID:AB_10563566) | - | 1:500 |
| goat-a-rat-488 (Thermo Fisher Scientific, A-11006, RRID:AB_2534074) | - | 1:500 |

### Protocol used for immunohistochemistry analysis

### Supplementary Table 3. Detailed protocol used for detection of different cell type markers and BrdU in cryosections of brain organoids.

|  | ms-a-Nestin | rb-a-GFAP | ms-a-NeuN | ms-a-MBP | rat-a-BrdU |
| --- | --- | --- | --- | --- | --- |
|  | rb-a-Sox2 | rb-a-Sox9 | rb-a-MAP2 | rb-a-Olig2 |  |
| Antigen retrieval | none | none | 10mM Tris + 0.1% Tween-20, pH 9, 25 min | 10mM Tris, 1mM EDTA + 0.05% Tween-20, pH 9, 20 min | 0.1M boric acid + 0.05% Tween-20, pH 8.5, 20 min |
| Blocking and permeabilisation solution | 10% Normal goat serum + 0.1 % Saponin in PBS | 10% Normal goat serum + 0.1 % Triton-X-100 in PBS | 10% Normal goat serum + 0.1 % Tween-20 in PBS | 10% Normal goat serum + 0.1 % Triton-X-100 in PBS | 10% Normal goat serum + 0.2 % Triton-X-100 in PBS |
| Primary antibody incubation solution | 1% Normal goat serum + 0.1 % Saponin in PBS  Over night | 1% Normal goat serum + 0.1 % Triton-X-100 in PBS  Over night | 1% Normal goat serum + 0.1 % Tween-20 in PBS  Over night | 1% BSA in PBS  Over two nights | 1% BSA + 0.1% Triton-X-100 in PBS  Over night |
| Secondary antibody incubation solution | 1% Normal goat serum + 0.1 % Saponin in PBS | 1% Normal goat serum + 0.1 % Triton-X-100 in PBS | 1% Normal goat serum + 0.1 % Tween-20 in PBS | 1% BSA + 0.1 % Triton-X-100 in PBS | 1% BSA + 0.1 % Triton-X-100 in PBS |
| Nuclear stain | deep red stain 1:1000 in PBS with 0.1% Saponin | deep red stain 1:1000 in PBS with 0.1% Triton | deep red stain 1:1000 in PBS with 0.1% Tween-20 | deep red stain 1:500 in PBS | deep red stain 1:500 in PBS with 0.1% Triton |
